# Lindoside, newly identified glycoside in linden honey using NMR spectroscopy

**DOI:** 10.1016/j.fochx.2026.104472

**Published:** 2026-09-14

**Authors:** Monika Škrjanc, Janez Plavec, Alen Albreht, Damjan Makuc

**Affiliations:** a Slovenian NMR Centre, National Institute of Chemistry, Hajdrihova 19, SI-1000 Ljubljana, Slovenia; b Faculty of Chemistry and Chemical Technology, University of Ljubljana, Večna pot 113, SI-1000 Ljubljana, Slovenia; c EN-FIST Centre of Excellence, Trg Osvobodilne fronte 13, SI-1000 Ljubljana, Slovenia; d Laboratory for Food Chemistry, Department of Analytical Chemistry, National Institute of Chemistry, Hajdrihova 19, SI-1000 Ljubljana, Slovenia

**Keywords:** Glycoside, Linden honey, NMR, Selective excitation, Diffusion ordered spectroscopy, Chemical marker

## Abstract

NMR is a powerful tool for the analysis of complex mixtures, such as honey, as it can identify relevant molecules without the need for isolation. NMR profiling of Slovenian honey revealed a linden-specific signal in the aliphatic region of the ^1^H NMR spectra that could not be attributed to any previously identified compound, prompting further structural characterization of a glycoside. The 2D NMR spectra showed that the aglycone part consists of a (2-hydroxyisopropyl)phenyl ring bound to a disaccharide moiety via ester bond. The disaccharide residue was determined to be β-gentiobiose based on diffusion-ordered spectroscopy and selective excitation NMR experiments. This study also establishes an NMR-based workflow for identifying glycosides in complex mixtures such as food. The identification of novel glycosides contributes to our understanding of the molecular composition of linden honey, to authentication of linden honey and provides a solid foundation for further studies of its potential biological functions.

## Highlights

• Power of NMR spectroscopy for elucidating glycoside structure in honey.
• Potential bioactive chemical marker of linden honey: lindoside.
• New insight into the chemical composition of linden honey.

## 1 Introduction

Honey is a natural bee product derived from nectar or honeydew, composed mainly of the monosaccharides glucose in its α and β forms and fructose as fructopyranose and fructofuranose. However, smaller proportions of other monosaccharides are also present, together with a wide range of other complex saccharides. Beyond sugars, honey is rich in other molecular groups, including organic acids, amino acids, proteins, enzymes, vitamins, minerals, phenolic compounds, terpenes, and esters. It is often used as a natural sweetener and exhibits a range of biological activities, including antifungal, antiviral, anti-inflammatory, antioxidant, anticancer, wound-healing and antibacterial activity (Bucekova et al., 2019; Nan et al., 2024; Sánchez et al., 2017; Scepankova et al., 2021; Tashkandi, 2021). Understanding the physiological role of these properties requires detailed chemical analysis, which can be challenging due to the complex and varying chemical composition of honey. This complexity is further increased by factors such as botanical origin, geographical region, climate, environmental conditions and beekeeping practices (Da Silva et al., 2016; Schievano et al., 2012).

Consumer interest in monofloral honey, motivated by its distinct organoleptic properties and health benefits, requires authenticity verification. However, traditional methods for honey analysis are often subjective and have limited capabilities. NMR offers a robust alternative, characterized by high reproducibility, speed, and simultaneous detection of multiple classes of compounds (Lolli & Caligiani, 2024). The key advantage of this method is its ability to determine the structure of unknown substances. These may be new adulterants that require monitoring or previously unknown honey constituents. Among the latter, chemical markers, molecules specific to a particular group of samples, such as a honey type, are especially important for NMR-based honey classification. Studying these markers improves general non-targeted analysis and determines the precise chemical composition of honey. Furthermore, it explains how specific compounds contribute to honey's properties, how environmental factors influence its composition, and helps determine differences in quality that may exist even between samples of the same type. To date, numerous honey-type specific chemical markers have been identified (Castro-Vázquez et al., 2014; Schievano et al., 2012; Truchado et al., 2008; Truchado et al., 2009). For example, kynurenic acid is a recognized chemical marker for chestnut honey (Consonni et al., 2019), while melezitose (Quirantes-Piné et al., 2024) and higher amounts of shikimic (Kivima et al., 2021) and citric acid (Keke & Cinkmanis, 2019) are present in honeydew honeys. For linden honey several markers have already been reported: carvacrol and α-terpinene (Juan-Borrás et al., 2014). Glycosides are also known chemical markers of the botanical origin of honey, with examples such as rutin in *Codonopsis* honey, which originates from plants widely used in traditional Asian medicine, and leptosin in manuka honey (Wang et al., 2022). Generally, glycosides consist of an aglycone linked to a sugar unit, typically d-glucose. The aglycone can be a molecule of various structural class, ranging from aliphatic to aromatic or steroidal structures, which contributes to structural diversity. In plants, the aglycone is conjugated to sugar unit in the endoplasmic reticulum by enzymes known as glycosyltransferases (Vogt & Jones, 2000). The sugar moiety is an important factor influencing their solubility, stability and bioavailability (Le Roy et al., 2016). Tiliaside, also referred to as lindenin glycoside is one of the known linden honey chemical markers, as it is present in significantly higher concentrations in linden honey compared to other types, and only in trace amounts in fir and chestnut honeys. Its aglycone is a terpene composed of a 1-(2-hydroxyisopropyl) 1,3-cyclohexadiene ring that is para-substituted with a carboxyl group, which forms an ester bond with the sugar moiety, β-gentiobiose (Frérot et al., 2006). In the same article they mentioned that NMR spectroscopy, in combination with UPLC/MS analysis, showed the presence of several glycosides of lindenin, which accounted for ca. 0.6 weight-% of the honey. Furthermore, two monoterpens were reported to be specific to linden honey: 4-(1-hydroxy-1-methylethyl)cy-clohexa-1,3-diene-1-carboxylic acid, which is a aglycone of tiliaside and 4-(1-methylethenyl)cyclohexa-1,3-diene-1-carboxylic acid (Frérot et al., 2006). It was recently shown that lindenin is absent in linden honeydew honey from Bosnia (Barbarić et al., 2025) as linden honey can originate from linden nectar or honeydew. Tiliaside exhibits antiparasitic activity in insects after hydrolysis. Specifically, a tiliaside-rich linden honey isolate reduces *Crithidia bombi* infections in bumblebees*, an effect that depends on* deglycosylation of glycoside in the insect gut releasing the active aglycone at the site of infection (Koch et al., 2022).

Honey is well known for its antibacterial properties, with the highest activity observed in chestnut and linden honey. In particular, the lowest MICs, highest biofilm inhibition, greatest membrane disruption against *P. aeruginosa, S. epidermidis*, and MRSA, membrane degradation, and anti-quorum-sensing activity were observed in every sample (Balázs et al., 2023; Farkas et al., 2022). This effect is attributed to general antibiotic factors found in honey, including hydrogen peroxide, physicochemical factors like low pH and high osmolarity (Kwakman & Zaat, 2012; Ogwu & Izah, 2025) as well as additional phytochemicals (Farkasovska et al., 2019; Luca et al., 2024). Among these, phenolic compounds have been shown to contribute to antibacterial activity (Bucekova et al., 2019), and it is possible that also aromatic glycosides play a role in this activity, as they are known to exhibit biological activity. For example, leptosin, an aromatic gentiobioside found in Manuka honey, has been identified as a key bioactive marker (Kato et al., 2012). The characterization of glycosides is important; however, their structural elucidation in honey remains challenging due to the susceptibility of the glycosidic bond to acid hydrolysis particularly in glycosyl esters. Additionally, analytical techniques often cannot detect or identify these compounds in their intact form (Cuyckens & Claeys, 2004). In this context, NMR offers the advantage of determining the detailed structure of molecules without the need for isolation, while NMR profiling can recognize honey type-specific spectral regions. For honey samples, these regions are found especially in the aromatic and aliphatic parts of ^1^H spectra. In linden honey samples, within these regions, signals assigned to known chemical marker tiliaside are observed, as well as additional low intensity signals which have not been identified and characterized. One of these unassigned ^1^H NMR resonances prompted the present investigation. According to Frérot et al. (2006) there are still uncharacterized glycosides in linden honey, therefore we expected to find previously unidentified glycoside. Newly identified compounds are conventionally isolated or synthesized for structural confirmation. In honey, however, the structural similarity between co-occurring glycosides and the complexity of the matrix make isolation impractical. If it were straightforward, these compounds would likely have been characterized already. The aim of this study was therefore to determine the structure of the intact glycoside, using solid-phase extraction as the only isolation step, by combining advanced NMR techniques – such as 2D correlation experiments, selectively excited 1D experiments, and DOSY – with MS as independent confirmation. We further hypothesized that this glycoside occurs at higher relative abundance in linden honey than in other botanical types, which we tested by ^1^H NMR profiling directly in the honey matrix, in the form that is relevant for authenticity assessment in practice.

## 2 Materials and methods

### 2.1 Sample preparation

Honey samples from different plant sources were obtained from the Slovenian Beekeepers' Association. 5 g of honey was dissolved in 5 mL of water and applied to a solid-phase extraction column Sep Pak C18 cartridge (Waters, Milford, Massachusetts, USA) preconditioned with 10 mL of methanol and 10 mL of water. Water (10 mL) was applied to the column and then mixtures of 700 μL water/methanol in various ratios (1:6, 2:5, 3:4, 4:3, 5:2, 6:1) were applied to the column sequentially. A 1D ^1^H NMR spectrum was recorded for each fraction and the best ratio between the signal intensity of the newly identified glycoside and the dominant tiliaside was observed in the 3:4 water/methanol mixture. This extract was further dried using centrifugal evaporator, then dissolved in 600 μL of deuterated water and used for detailed NMR studies.

### 2.2 NMR analysis

1D and 2D NMR spectra were acquired on 600 MHz Bruker Avance NEO spectrometer equipped with Quadruple-resonance, Carbon-optimized, Inverse-detection probe (QCI probe) (Bruker, Rheinstetten, Germany). All experiments were performed at 25 °C. The 1D and 2D spectra were apodized with an exponential, sine-squared or cosine-squared function and zero filled before the Fourier transformation. NMR data were processed using MestReNova NMR software (Mestrelab Research, Santiago de Compostela, Spain).

The 1D proton experiment (Fig. S1) was run using noesygppr1d pulse sequence with 128 scans, 10.0 s relaxation delay and 64 K data points in the time domain to cover a spectral width of 15.1 ppm. To assess the reproducibility of the NMR measurements and the stability of the signal assignments, we repeated the measurements of the same sample at least four times. In addition, 1D ^1^H NMR spectra were acquired from independently prepared SPE extracts, including extracts from different honey samples. For repeated measurements of the same sample, the chemical shifts were identical. Across independently prepared extracts, including those obtained from different honey samples, the chemical shifts of the relevant signals were consistent, with differences of no more than 0.04 ppm. These observations confirm the good reproducibility of the NMR measurements and support the reliability of the signal assignments.

2D ^1^H–^13^C HSQC-TOCSY spectra (Figs. S4 and S5) were measured using hsqcdietgpsisp.2 pulse sequence with spectral widths of 16.0 ppm and 165.0 ppm for the ^1^H- and ^13^C-dimensions, respectively, 2 K data points for the ^1^H-dimension, a relaxation delay of 1.5 s, 128 scans, 192 increments and mixing time 20–120 ms, incremented by 20 ms.

2D ^1^H–^13^C HMBC spectrum (Fig. S3) was acquired using hmbcetgpl3nd pulse sequence with spectral width of 13.0 ppm and 220.0 ppm for ^1^H- and ^13^C-dimensions, respectively, 4 K data points for ^1^H-dimension, relaxation delay 2.0 s, 128 scans and 192 increments.

Pseudo 2D DOSY spectrum was acquired using ledbpgppr2s pulse sequence with spectral width of 23.0 ppm, 16 K data points for ^1^H-dimension, a relaxation delay of 1.5 s and 128 scans. For DOSY gradient array 16 different gradient strengths were incremented from 0.963 to 47.187 G/cm using diffusion delay (big delta) of 30 ms and gradient pulse duration (little delta) of 4 ms.

1D selective TOCSY for sugar residue B of lindoside was acquired using seldigpzs sequence with spectral width of 19.8 ppm, 6 K data points, a relaxation delay of 1.0 s and 128 scans. The H1′ resonance at *δ*_*H*_ 5.771 ppm was selectively excited using a Gaussian 180° shaped pulse with a duration of 150 ms, followed by a DIPSI2 pulse train with mixing times in the range from 20 to 120 ms, incremented by 20 ms.

1D selective TOCSY-NOESY experiment for lindoside was acquired using stepnoesyzs sequence with 15,232 scans, a relaxation delay of 1.0 s, 6 K data points and spectral width of 19.8 ppm. The shapes of the 180° selective excitation pulses for the 1D selective TOCSY-NOESY experiment were both Gaussian. For TOCSY pulse sequence element the duration of the pulse was set to 80 ms, mixing time was set to 120 ms and H1′ at *δ*_*H*_ 5.766 ppm was selectively excited. For NOESY pulse sequence element the duration of the pulse was set to 66 ms, mixing time was set to 600 ms and H6b′ at *δ*_*H*_ 3.827 ppm was selectively excited.

1D selective TOCSY-NOESY experiment for tiliaside (Fig. S7a) was acquired using stepnoesyzs sequence with 236 scans, a relaxation delay of 1.0 s, 6 K data points and spectral width of 19.8 ppm. The shapes of the 180° selective excitation pulses for the 1D selective TOCSY-NOESY experiment were both Gaussian. For TOCSY pulse sequence element the duration of the pulse was set to 80 ms, mixing time was set to 120 ms and H1′ at *δ*_*H*_ 5.588 ppm was selectively excited. For NOESY pulse sequence element the duration of the pulse was set to 66 ms, mixing time was set to 600 ms and H6b′ at *δ*_*H*_ 3.804 ppm was selectively excited.

1D selective TOCSY for sugar residue C of lindoside (Fig. S6) was acquired using seldigpzs sequence with spectral width of 19.8 ppm, 6 K data points, a relaxation delay of 1.0 s and 256 scans. The H1′ resonance at *δ*_*H*_ 4.413 ppm was selectively excited using a Gaussian 180° shaped pulse with a duration of 150 ms, followed by a DIPSI2 pulse train with mixing times 20, 40, 60, 80 and 120 ms.

### 2.3 LC–HRMS/MS analysis

Honey extracts were diluted 500-fold with 20% MeOH (*v/v*) and transferred to autosampler vials and maintained at 10 °C until analysis. LC–HRMS/MS analysis was performed using a Waters Acquity Premier liquid chromatograph coupled to a Synapt XS high-resolution mass spectrometer equipped with an electrospray ionisation source. Chromatographic separation was achieved on a Waters Acquity Premier BEH C18 column (50 × 2.1 mm i.d., 1.7 μm) maintained at 40 °C*. mobile* phase A consisted of 0.01% formic acid, while mobile phase B consisted of acetonitrile containing 0.01% formic acid. The following gradient programme was applied: 0–8 min (2% B), 8–15 min (2–20% B), 15–17 min (20–80% B), 17–17.1 min (80–2% B), 17.1–20 min (2% B). The flow rate was 0.6 mL min^−1^ and the injection volume was 10 μL. Mass spectra were acquired in negative-ion electrospray ionisation mode over an *m/z* range of 50–1300. The principal ion-source parameters were as follows: spray voltage, 2 kV; source temperature, 120 °C; desolvation temperature, 550 °C; nebulising gas, 5 bar; cone gas flow, 50 L/h; and desolvation gas flow, 800 L/h. The instrument was externally calibrated using sodium formate calibration solution while leucine enkephaline lock masses at *m/z* 236.1064 and 554.2672 were used during acquisition. Product-ion spectra were acquired using targeted MS/MS on the precursor ion at *m/z* 503.1764 with the collision energy set at 30 V. MS/MS spectra were recorded over *m/z* 50–1200. Raw data were processed using MassLynx V4.2 software (Waters). Extracted-ion chromatograms were generated using a mass tolerance of 0.05 mDa. Elemental compositions were assigned from the accurate precursor and product-ion masses, mass errors, isotope distributions and applicable elemental constraints. Putative compound annotations were based on chromatographic behaviour, accurate mass, isotope pattern and diagnostic MS/MS product ions.

## 3 Results and discussion

1.1. ^1^H NMR identified glycoside with aromatic aglycone

**Fig. 1:**
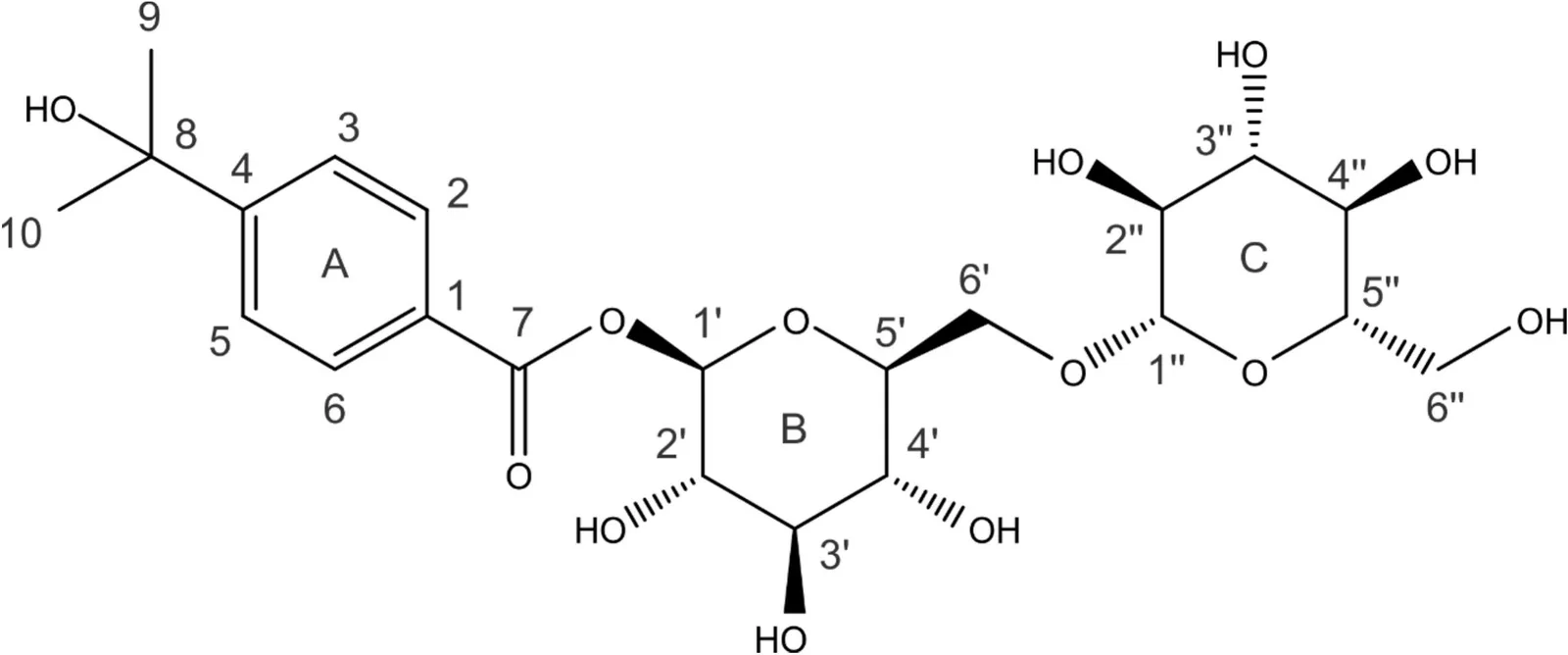
Chemical structure of β-D-gentiobios-1-yl 4-(2-hydroxyisoprop-2-yl) benzoate, lindoside. The structure consists of three main parts: the aglycone part (A), the first sugar residue (B) and the second sugar residue (C). Atom numbering used in structure elucidation is shown.

NMR profiling of seven types of Slovenian honey revealed linden honey-specific regions in ^1^H NMR spectrum of linden honey extract, which are labelled with grey areas in Fig. S1. These regions predominantly exhibit signals attributed to the known tiliaside, alongside additional signals with low intensity that cannot be assigned to any previously known molecule described in the literature. Therefore, NMR-based structural elucidation was performed in order to characterize the unassigned signals. Minor compounds may be undetectable in the 2D NMR spectra due to the intense sugar signals present in honey samples. To overcome this dynamic range limitations and suppress intense signals of sugars solid-phase extraction (SPE) was performed on representative sample of linden honey. In the ^1^H NMR spectrum of the honey extract the aliphatic region still exhibited extensive overlap of signals from sugar units, but several distinct resonances of comparable intensity were detected in the less crowded part of spectrum. Upon initial inspection, two aromatic proton signals at *δ*_*H*_ 8.034 (H2/H6) and 7.581 ppm (H3/H5), an anomeric doublet H1′ at *δ*_*H*_ 5.767 ppm as well as an aliphatic singlet at *δ*_*H*_ 1.515 ppm were observed. These signals suggest that they originate from a more complex molecule, presumably a phenyl moiety bound to a sugar, consistent with the structure of glycosides. A detailed NMR analysis described below confirmed that the signals correspond to a glycoside, that we named lindoside, with the chemical structure shown in Fig. 1.

### 3.1 DOSY data supporting the molecular system consisting of aglycone and two sugar units

The size distribution of the molecules in linden honey extract was investigated using DOSY NMR. This technique separates molecules based on their diffusion coefficients, which are inversely proportional to molecular size as described by the Stokes-Einstein equation (Morris, 2009).

**Fig. 2:**
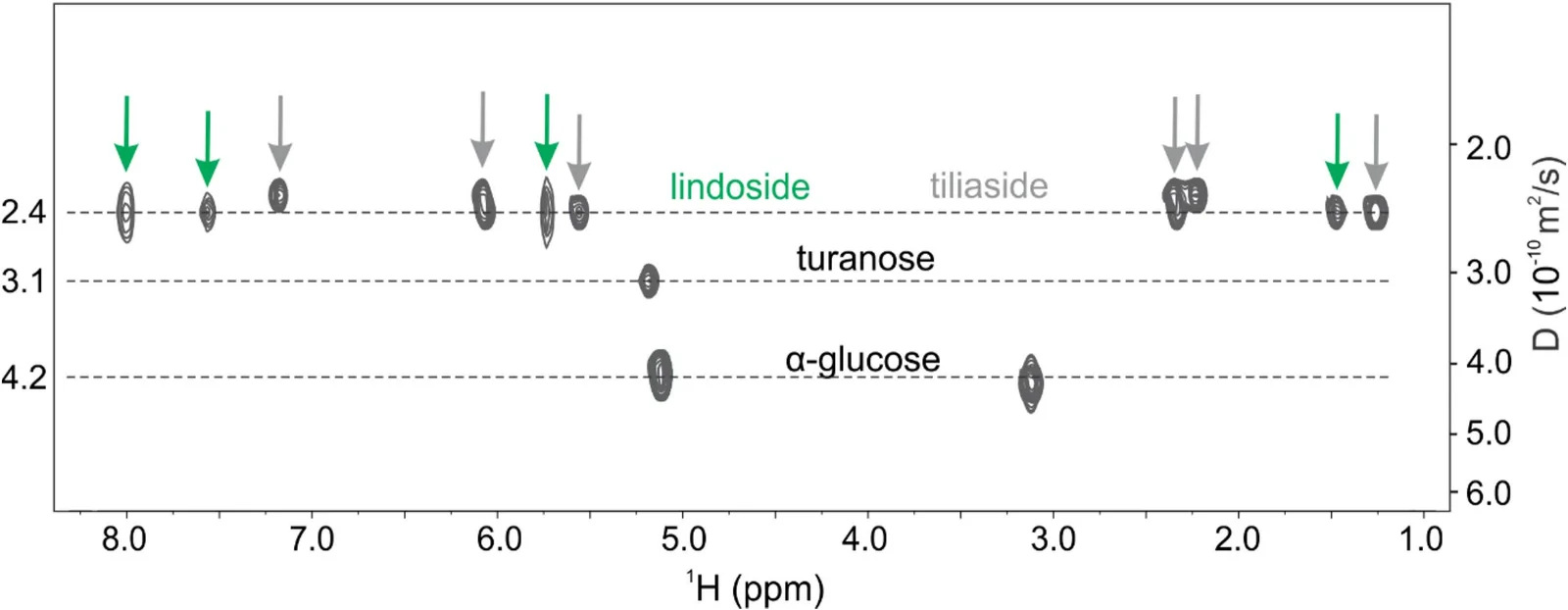
2D ^1^H DOSY spectrum of linden honey extract dissolved in D_2_O, to which a small amount of the original honey solution was added. Diffusion coefficients of lindoside (green arrows) were determined as well as diffusion coefficient of turanose, α-glucose and tiliaside (grey arrows), which were used as internal standards. The measured values are labelled with dotted lines. (For interpretation of the references to colour in this figure legend, the reader is referred to the web version of this article.)

Glycosides can be easily distinguished from other molecules in the sample in the DOSY spectrum because they are larger compared to the monosaccharides and disaccharides found in honey. In the DOSY spectrum of linden honey shown in Fig. 2, lindoside signals exhibit a diffusion coefficient of approximately 2.4 × 10^−10^ m^2^/s (green arrows, Fig. 2). The same value was determined for tiliaside (grey arrows, Fig. 2). This indicates that lindoside exhibits a similar molecular structure, consisting of aglycone moiety and two sugar units. The proposed number of structural units in lindoside is consistent with the trend observed for glycosides in honey, which typically contain more than one sugar unit, predominantly two or three.

Recognizing that, for small molecules, the Stokes–Einstein equation does not reliably relate the diffusion coefficient to molecular weight because it assumes that the fluid through which molecules diffuse is a continuum and that the molecules of interest are hard spheres, we note that these assumptions are usually invalid for small molecules. This is why we used an internal calibration method to interpret the measured diffusion coefficients quantitatively (Evans, 2020). For this purpose, a small amount of the parent honey sample was added to the SPE extract. This ensured the presence of two internal standards α-glucose (monosaccharide) and turanose (disaccharide), which were previously identified in our Slovenian linden honey samples.

The diffusion coefficient for the three calibrants and for lindoside were determined from the same DOSY experiment performed on one sample, therefore the viscosity, solvent and temperature were the same for all analytes. Linear regression was performed on logD of logM_w_ according to the power-law relation log D = α · log M_w_ + logK and it resulted in relationship logD = −0.535 · log M_w_ – 8.17 with R^2^ = 0.992. The exponent corresponds to the one reported for proto-quercitol in honey water solution (α = −0.46) (Gresley et al., 2012). Residuals of the calibrants correspond to at most 5.2% on the molecular weight scale. Using this calibration to estimate molecular weight from diffusion coefficient of lindoside signal gives 523 g/mol, consistent with the value calculated for the proposed structure (504.2 g/mol) and the fact that lindoside has three units (aglycone + two sugar units).

### 3.2 Aglycone structure determination by 2D HMBC spectrum

**Fig. 3:**
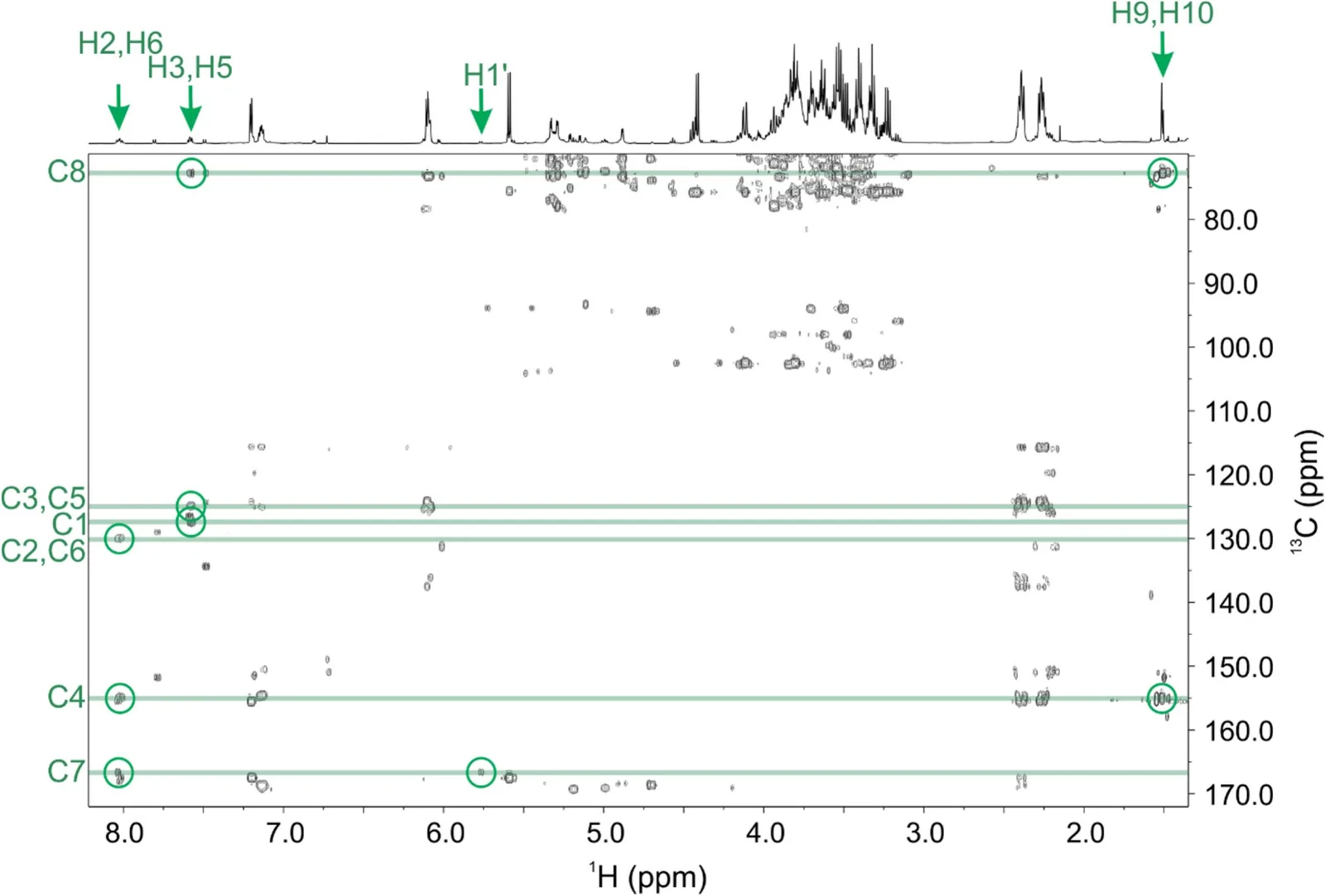
Region of the 2D ^1^H–^13^C HMBC spectrum of linden honey extract dissolved in D_2_O. The key long-range correlation signals characterizing the substituents attached to the phenyl ring A are marked with green circles, with green lines further indicating the associated ^13^C signals; the numbers correspond to the atom numbering shown in Fig. 1. (For interpretation of the references to colour in this figure legend, the reader is referred to the web version of this article.)

The ^1^H–^13^C long-range correlations detected in the HMBC experiment were used to identify the substituent groups bonded to the phenyl ring (Fig. 3). The observed correlation signals between the H9/H10 methyl groups at *δ*_*H*_ 1.515 ppm and the C4 carbon atom at *δ*_*C*_ 154.9 ppm, as well as between the aromatic H2/H6 protons at *δ*_*H*_ 8.034 ppm and the C4 carbon confirmed that the 2-hydroxyisopropyl group is attached to the carbon atom at position 4. Furthermore, the H2/H6 protons exhibited HMBC correlation signal with the C7 carboxyl carbon at *δ*_*C*_ 166.7 ppm, which in turn correlated with a doublet at *δ*_*H*_ 5.767 ppm assigned to H1′ proton. This suggests that ester group in para position to the 2-hydroxyisopropyl group links the sugar ring B and aglycone.

### 3.3 Identification of the first sugar unit by selective TOCSY and HSQC-TOCSY

Further structural characterization was challenging due to the condensed region of the ^1^H and ^13^C NMR chemical shifts corresponding to the sugar moieties. The well-resolved signal of the anomeric H1′ proton at *δ*_*H*_ 5.767 ppm enabled application of selective NMR experiments combined with magnetization transfer through bond and through space. This method facilitated selective observation and identification of the desired correlation signals.

**Fig. 4:**
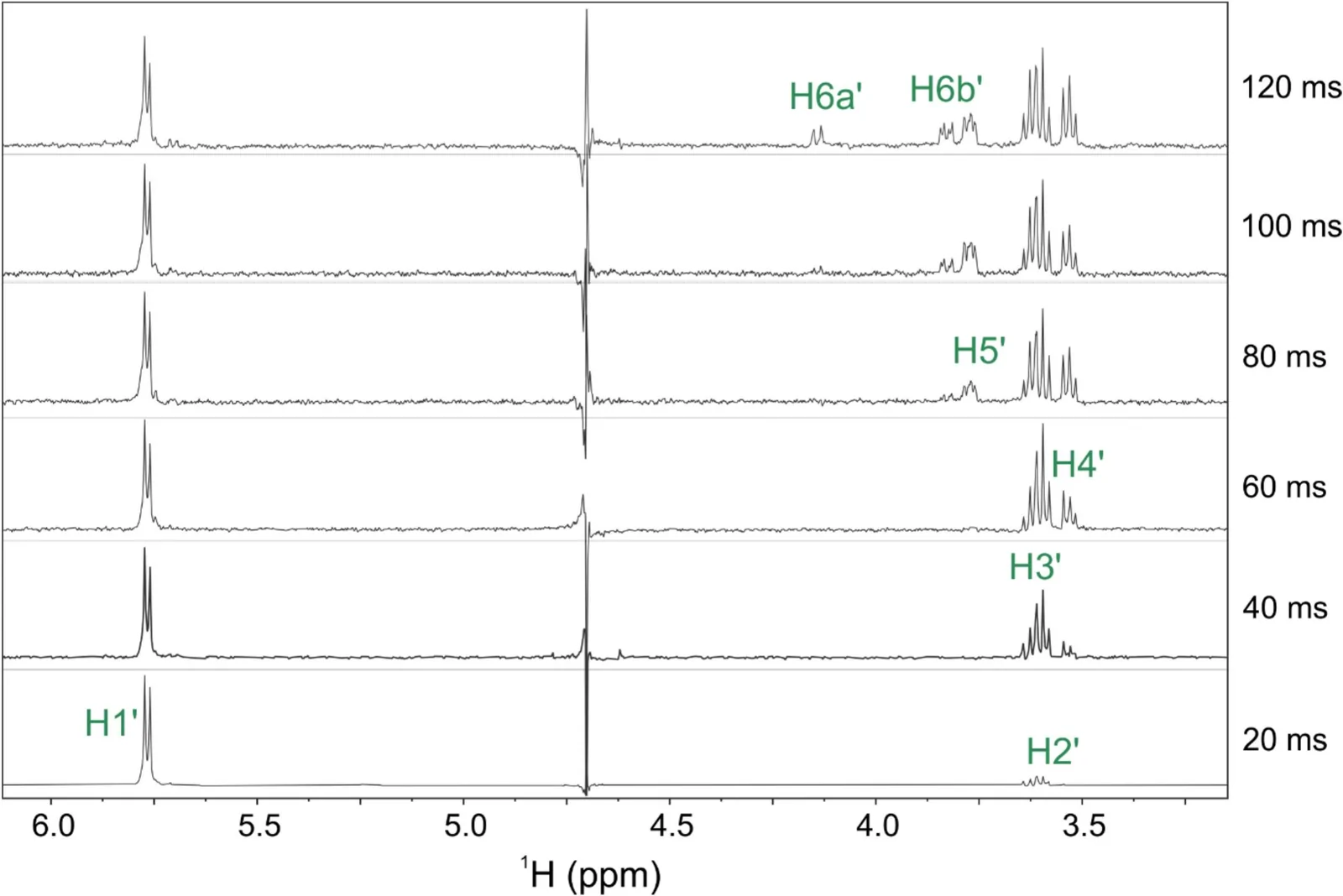
Sugar region of the stacked 1D ^1^H selective TOCSY spectra of linden honey extract dissolved in D_2_O recorded by selective excitation of the H1′ proton and using various mixing times in the range from 20 to 120 ms, with the assigned protons of sugar unit B.

A TOCSY experiment with magnetization transfer through bonds was performed to distinguish all proton signals within the sugar unit B (Fig. 4). By adjusting the mixing time in the range from 20 to 120 ms, the extent of magnetization transfer was controlled to follow coupling interactions between the selectively excited H1′ proton and protons within the same spin system. The NMR chemical shifts, multiplicities and *J*-coupling constants of sugar unit B protons were determined and are reported in Table 1. The proton-proton *J*-coupling constants observed throughout the sugar unit B are consistent with the stereochemistry of a glucose residue. The H1′ proton exhibits proton-proton *J*-coupling constant of 7.8 Hz with its neighbouring H2′ proton, indicating diaxial orientation of H1′ and H2′ according to the Karplus equation (Coxon, 2009). This implies that the ester linkage on the same C1′ atom as anomeric proton is equatorially oriented, which confirms a β-anomeric configuration. To assign the carbon chemical shifts of sugar unit B, a 2D ^1^H–^13^C HSQC-TOCSY experiment, which correlates protons to directly bonded carbons, was recorded with various mixing times and is shown in Fig. S4.

**Table 1:** ^1^H and ^13^C NMR chemical shifts, multiplicity and coupling constants of lindoside signals.

Position	*δ*_*H*_ (ppm), multiplicity, *J* (Hz)	*δ*_*C*_ (ppm)
1	–	127.4
2, 6	8.03 (d, *J* = 8.8)	129.9
3, 5	7.58 (d, *J* = 8.8)	124.9
4	–	154.9
7	–	166.7
8	–	72.8
9, 10	1.51 (s)	30.2
1′	5.77 (d, *J* = 7.8)	94.3
2′	3.59 (dd, *J* = 8.9, 7.8)	71.9
3′	3.63 (dd, *J* = 9.1, 8.9)	75.3
4′	3.53 (dd, *J* = 9.1)	68.9
5′	3.77 (dd, *J* = 9.1, 5.3)	75.8
6′	4.14 (d, *J* = 11.5), 3.83 (dd, *J* = 11.5, 5.3)	68.1
1″	4.41 (d, *J* = 8.0)	102.6
2″	3.23 (m)	73.0
3″	3.41 (dd, J = 8.9)	75.7
4″	3.31 (m)	69.4
5″	3.33–3.37 (m)	76.2
6″	3.82 (d, *J* = 12.5), 3.63 (dd, *J* = 12.5, 5.5)	60.7

### 3.4 Assignment of the second sugar unit

**Fig. 5:**
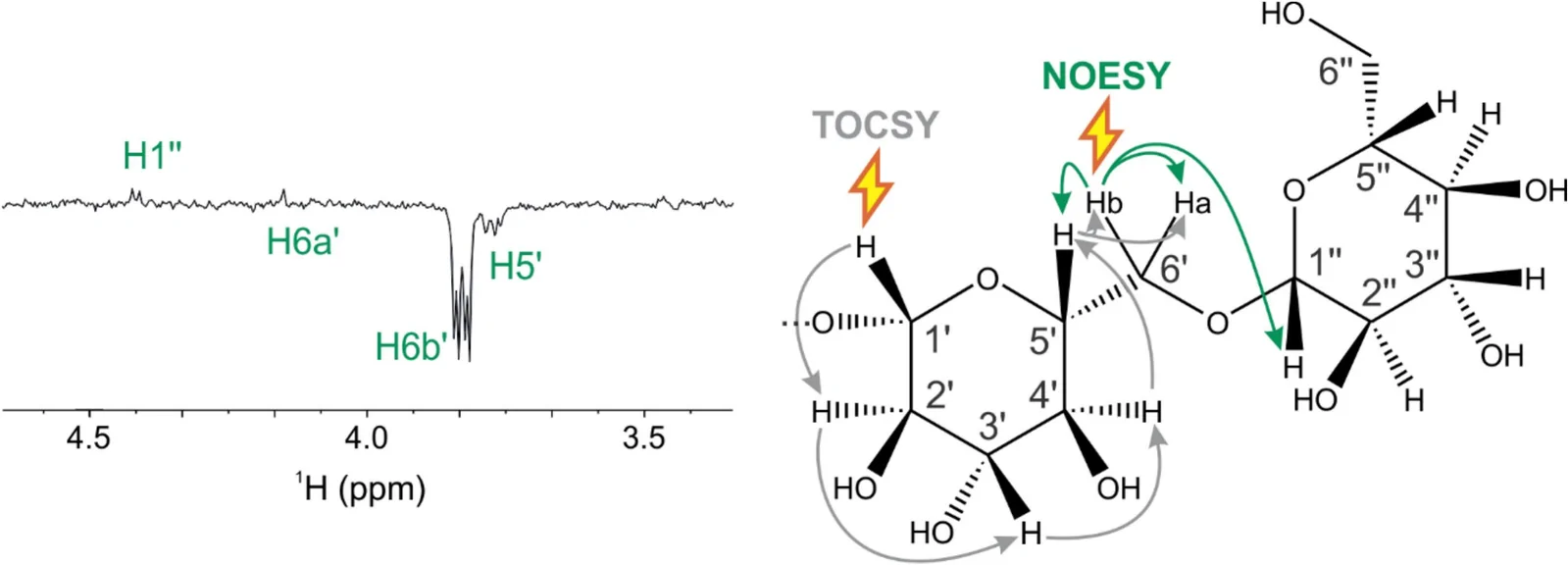
The NOE enhancements between selectively excited H6b′ and H5′, H6a′ and H1″ protons observed in the selective TOCSY-NOESY spectrum. In the schematic illustration the magnetization transfer throughout molecule during preparation step is labelled with grey colour whereas the resulting NOE enhancements are labelled with green. (For interpretation of the references to colour in this figure legend, the reader is referred to the web version of this article.)

Further magnetization transfer from selectively excited H1′ proton through bonds in the molecule was interrupted by the glycosidic oxygen atom. Therefore, a selective TOCSY-NOESY experiment was used to detect an NMR correlation signal between sugar units B and C. While the H1′ signal is not the final detected peak of interest, its selective excitation using TOCSY experiment was a preparation step because all other sugar protons were present in the severely overlapped sugar region. The second step of experiment was selective NOESY where magnetization was transferred through dipole-dipole interactions between protons in spatial proximity even across oxygen atom. During this experiment H6b′ proton at *δ*_*H*_ 3.827 ppm was selectively excited, which revealed NOE enhancements of H5′, H6a′ and H1″ protons (Fig. 5). The doublet of the anomeric H1″ proton at *δ*_*H*_ 4.413 ppm in the resulting spectrum confirms the presence of a second sugar unit. Its *J*-coupling constant with neighbouring H2″ proton of 8.0 Hz indicates a diaxial orientation of protons and suggests that sugar unit C exhibits a β-anomeric configuration and thus confirms a β(1,6)-glycosidic bond between sugar units B and C.

To determine the structure of sugar unit C, additional selective TOCSY experiments were performed by selectively exciting the H1″ resonance at *δ*_*H*_ 4.413 ppm in the severely overlapped sugar region (Fig. S6). The H1″ anomeric proton signal of lindoside coincides with H1″ anomeric proton signal of tiliaside, which is demonstrated in the Fig. S7 with a comparison of the selective TOCSY–NOESY spectra of both compounds. Therefore, both protons were selectively excited during the experiment. In the recorded spectrum single TOCSY spin system was observed which suggests that signals of lindoside sugar units C are overlapped with signals of tiliaside sugar units C. The proton-proton *J*-coupling constants observed throughout the spin system are consistent with the stereochemistry of a glucose residue. The overlapping signals of both molecules arise from the identical chemical environment of sugar units C, because the molecular structures are very similar (both contain β-gentiobiose). If the sugar units were different, more pronounced differences would be expected, since even small changes in the sugar moiety, such as α- and β-configuration, would produce a different set of NMR signals for the entire spin system. Moreover, the presence of the second sugar unit and the number of its atoms were confirmed by the previously described DOSY and MS data. However, this step represents a limitation that we encountered during the structural determination of lindoside by NMR.

### 3.5 Quantitative characterization

**Fig. 6:**
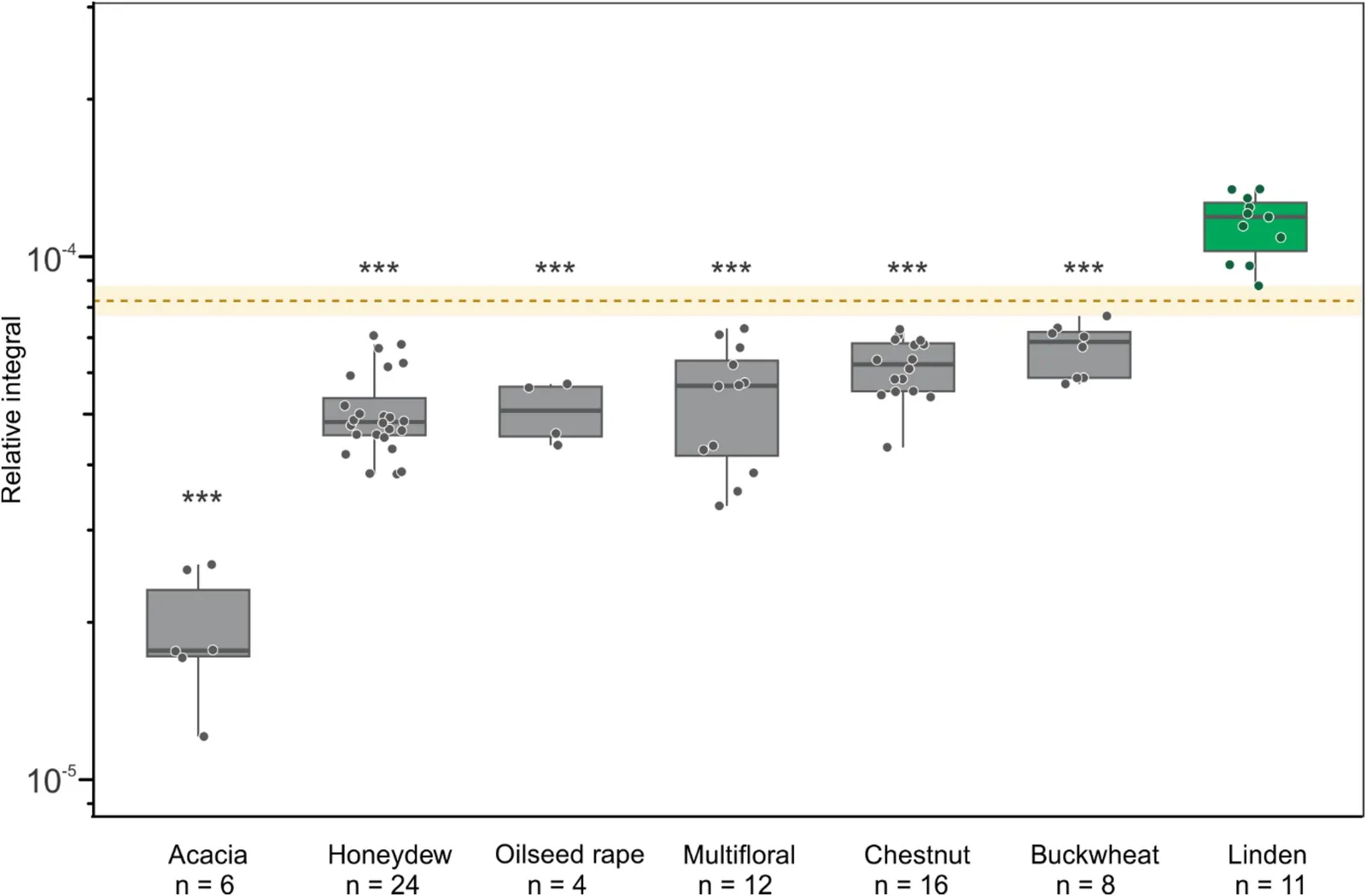
Relative integral of the signal at δ 1.58 ppm (integration region *δ* 1.5369–1.5966 ppm) in 81 Slovenian honey samples from seven botanical types. Boxes show the median and interquartile range, whiskers represent 1.5 × IQR, and points denote individual samples. ***p < 0.001 versus linden, calculated with Tukey HSD on ln-transformed values. The orange dashed line indicates the classification threshold, and the shaded orange band shows the separation gap between linden and all other types.

NMR profiling of 81 Slovenian honey samples, information on which can be found in the supplementary material, revealed statistically relevant bins after PCA analysis that separated linden samples from non-linden samples. In one of them, in the integration region δ 1.5369–1.5966 ppm, a signal assigned to the 2-hydroxyisopropyl group of lindoside was observed. The distribution of relative integrals among samples of different honey types is shown in the box plot in Fig. 6. The relative integral of this region differentiates between linden and non-linden samples. Together with the known chemical markers, it is a valuable feature for determining the authenticity of linden honey using NMR profiling, which is a promising technique for differentiating honey types.

The story becomes complicated because this signal shows HMBC correlations to a cluster of signals with similar chemical shifts. They can most likely be assigned to structurally similar molecules that share the same aglycone, which is bound to different disaccharide moiety. Because this cluster of molecules, in which lindoside is the predominant species, is present at low concentrations, correlations between the aglycone CO group and other sugar protons in the molecule are not visible in the HMBC spectrum. It is only present for this cluster in lindoside, and the aim cannot be confirmed. LC–HRMS analysis provided further evidence for multiple lindoside isomers in the honey extract (Figs. S9–S11). The three most abundant each produced a deprotonated molecular ion, [M − H]^−^, at *m/z* 503.1764, consistent with the elemental composition C₂₂H₃₁O₁₃^−^, and closely similar MS/MS spectra containing diagnostic product ions at *m/z* 179.0687, assigned to the aglycone, and 341.1078, assigned to the sugar moiety (Fig. S11). Together, the accurate-mass and fragmentation data support their tentative identification as lindoside isomers. However, the same phenomenon is observed for tiliaside in the HMBC spectrum, where correlations of the aglycone to different sugar protons are visible (Fig. S8), confirming the presence of isomers.

### 3.6 Significance of lindoside structural characterization for linden honey authentication and specific features

The structural characterization of lindoside is deemed important for the broadening of scientific knowledge on the chemical composition of honey. Lindoside is found in lindenhoney-specific regions of 1D ^1^H NMR spectra and could be a chemical marker of linden honey. Chemical markers are important for honey authenticity because they enable the precise classification of botanical sources and possible assessment of mixtures, overcoming the limitations of traditional methods that may be subjective. They can contribute to linden honey-specific features such as taste, odor, and biological activity. Structural characterization with NMR presents the foundation for quantitative analysis, which is important for molecules with potential biological activity. Glycosides are promising candidates for bioactive compounds because their sugar moiety increases the solubility of an otherwise poorly soluble aglycone. Furthermore, sugar moiety stabilizes the aglycone's inactive form, contributing to safer use and efficiency at the site of infection. For tiliaside, antiparasitic activity of its aglycone after cleavage of the glycosidic bond was shown in bumblebees. Because of the structural similarity between lindoside and tiliaside, isolating one without the other is difficult. Lindoside may also possess antiparasitic activity, which would need to be further tested.

We also aimed to demonstrate an efficient NMR workflow for identification of glycosides in complex mixtures without the need for complementary methods such as MS. NMR offers various techniques, such as translational diffusion measurements and selective excitation experiments that were used to unambiguously determine the structure of the glycoside in intact form in a complex mixture. Establishing this structure is the foundation for exploring its biological function, planning biological activity assays, and determining its role in linden honey-specific properties.

## 4 Conclusions

The comprehensive NMR spectroscopy study, including 2D and selective excitation techniques, enabled us to elucidate the structure of lindoside in Slovenian linden honey. Diffusion-ordered spectroscopy (DOSY) indicated that the molecular size of lindoside is comparable to that of the previously known tiliaside. The aglycone was identified as a 2-hydroxyisopropyl-substituted phenyl moiety, and its ester linkage to the anomeric proton of the first sugar unit was detected using a long-range 2D HMBC NMR spectrum. Due to the signal overlap in the sugar region, selective excitation of the anomeric protons was used for determination of the structural details of sugar part of molecule. Selective TOCSY confirmed the first sugar unit as β-glucose. The presence of the second sugar unit was confirmed by detecting its anomeric signal in the selective TOCSY-NOESY spectrum and further supported by the DOSY results. To fully characterize the spin system of this second sugar unit C, a further selective TOCSY experiment was recorded, in which H1″ protons of lindoside and tiliaside were simultaneously excited because their signals fully overlap. Based on the signals observed in selective TOCSY spectra sugar unit C of lindoside is a β-glucose moiety. The absence of any other signal in the spectrum indicates that all proton signals of sugar unit C of lindoside completely overlap with those of tiliaside sugar unit C, due to the identical structure of this unit. This step remains the principal limitation of the assignment.

The results and structural elucidation of lindoside is important for several reasons. Firstly, its higher concentrations in linden honey compared to other types represent a new identified chemical marker for verifying honey's botanical origin and quality. Secondly, the identification of lindoside contributes to a better understanding of the complex chemical composition of linden honey. Thirdly, as glycosides often exhibit biological activity, this newly identified molecule is a candidate for future studies to elucidate specific characteristics of linden honey, including its bioactivity. Finally, the study confirms that the applied NMR workflow is a powerful tool for analyzing specific components in honey.

## CRediT authorship contribution statement

**Monika Škrjanc:** Writing – original draft, Visualization, Methodology, Investigation, Formal analysis, Data curation. **Janez Plavec:** Writing – review & editing, Resources, Project administration, Methodology, Funding acquisition, Conceptualization. **Alen Albreht:** Formal analysis, Writing – original draft. **Damjan Makuc:** Writing – review & editing, Validation, Supervision, Project administration, Methodology, Formal analysis, Conceptualization.

## Funding

This work was financially supported by the Slovenian Research and Innovation Agency (ARIS) (grant number P1-0242).

## Declaration of competing interest

The authors declare that they have no known competing financial interests or personal relationships that could have appeared to influence the work reported in this paper.

## Data Availability

The NMR spectroscopy data that support the findings of this study are available under Accession DOI: 10.17632/5cdpbpbkcz.1.

## Appendix A Supplementary data

Supplementary material Supporting NMR and LC-HRMS/MS data for the identification and structural characterization of lindoside in linden honey, including detailed NMR spectra, NMR profiling data, and LC-HRMS/MS analysis.
