# Supplementary material for: Lindoside, newly identified glycoside in linden honey using NMR spectroscopy
Source: Food Chem X. 2026 Sep 14;39:104472. doi: 10.1016/j.fochx.2026.104472 (PMC13599650; doi:10.1016/j.fochx.2026.104472)
Supplement: Supplementary file 1 [file mmc1.docx]

**Supplementary material**

**Lindoside, newly identified glycoside in linden honey using NMR spectroscopy**

Monika Škrjanc^a,b^, Janez Plavec^a,b,c^, Alen Albreht^d^, Damjan Makuc^a*^

^a^ Slovenian NMR Centre, National Institute of Chemistry, Hajdrihova 19, SI-1000 Ljubljana, Slovenia

^b^ Faculty of Chemistry and Chemical Technology, University of Ljubljana, Večna pot 113, SI-1000, Ljubljana, Slovenia

^c^ EN- FIST Centre of Excellence, Trg Osvobodilne fronte 13, SI-1000 Ljubljana, Slovenia

^d^ Laboratory for Food Chemistry, Department of Analytical Chemistry, National Institute of Chemistry, Hajdrihova 19, SI-1000 Ljubljana, Slovenia

^*^ Corresponding author

Table of Contents

[**1.**	**Proton NMR spectrum**	1](#_Toc239012639)

[**2.**	**DOSY**	2](#_Toc239012640)

[**3.**	**Full structural assignment**	4](#_Toc239012641)

[**4.**	**Comparison of TOCSY-NOESY NMR spectra of lindoside and tiliaside compounds**	8](#_Toc239012642)

[**5.**	**NMR profiling information and quantification data for lindoside**	9](#_Toc239012643)

[**6.**	**LC–HRMS/MS analysis**	14](#_Toc239012644)

[**7.**	**References**	16](#_Toc239012645)

Table of Figures

[**Figure S1**. 1D ^1^H spectrum of linden honey extract dissolved in D_2_O. The well-resolved signals of tiliaside are labelled with orange arrows, signals of new glycoside lindoside are labelled with green arrows. Linden honey specific regions obtained with PCA performed during NMR profiling of Slovenian honey are marked with grey bands.	1](#_Toc239061772)

[**Figure S2.** Calibration curve relating molecular weight and diffusion coefficients of internal standards.	3](#_Toc239061773)

[**Figure S3.** 2D ^1^H-^13^C HMBC spectrum of linden honey extract dissolved in D_2_O.	4](#_Toc239061774)

[**Figure S4.** Sugar region of H1' proton in the stacked 2D ^1^H-^13^C HSQC-TOCSY spectra of linden honey extract dissolved in D_2_O with arrayed mixing time (20–120 ms), incremented by 20. Carbon chemical shifts of sugar unit B are labelled with green lines.	5](#_Toc239061775)

[**Figure S5.** 2D ^1^H-^13^C HSQC-TOCSY spectrum of linden honey extract dissolved in D_2_O with mixing time 100 ms.	6](#_Toc239061776)

[**Figure S6.** Sugar region of stacked 1D ^1^H selective TOCSY spectra of linden honey extract dissolved in D_2_O recorded by selectively exciting the H1” proton and using various mixing times (20, 40, 60, 80 and 120 ms) with assigned protons of sugar unit C.	7](#_Toc239061777)

[**Figure S7.** 1D ^1^H selective TOCSY-NOESY spectra of linden honey extract dissolved in D_2_O with assignment of H5', H6a', H6b' and H1” protons of tiliaside (a) and lindoside (b). The chemical structures of tiliaside (c) and (d) lindoside are shown, with main structural difference highlighted by magenta arrows.	8](#_Toc239061778)

[**Figure S8.** Region of the 2D HMBC spectrum of linden honey extract dissolved in D_2_O. Key long-range correlation signals characterizing the substituents attached to phenyl ring A are marked with green circles; the numbers correspond to the atom numbering shown in Figure 1. Green lines show correlations of the cluster of signals with similar chemical shifts, assigned to molecules structurally related to lindoside, whereas orange lines mark the corresponding set for tiliaside. Orange arrows show correlations between aglycone signals and additional sugar signals.	13](#_Toc239061779)

[**Figure S9.** Extracted ion chromatogram (m/z 503.1764) of honey extract, diluted 500-fold with 20% MeOH. Major lindoside derivatives were found at the following retention times: 10.04 min, 11.04 min, and 11.16 min.	14](#_Toc239061780)

[**Figure S10.** A representative lindoside derivative MS spectrum acquired at retention time 10.1 min. The spectrum was acquired in negative ionization mode.	15](#_Toc239061781)

[**Figure S11.** MS/MS spectra of major lindoside derivatives found at 10.04 min (top), 11.04 min (middle), and 11.16 min (bottom). In all cases, a precursor ion at m/z 503.1764 was fragmented. The spectra were acquired in negative ionization mode.	15](#_Toc239061782)

Table of Tables

[**Table S1.** Molar mass and diffusion coefficients of internal standards used for the calibration curve and newly characterized lindoside. DOSY spectrum recorded on the same sample using solvent D_2_O, T = 25°C. Calculated M_W_ values obtained from the calibration curve are reported together with the deviation from their actual M_W_ in %.	3](#_Toc239012657)

[**Table S2.** Linest function results	3](#_Toc239012658)

[**Table S3.** List of samples that were used for NMR profiling	9](#_Toc239012659)

[**Table S4.** Relative integral (1.5369-1.5966 ppm) by honey type	12](#_Toc239012660)

[**Table S5.** Overall tests	12](#_Toc239012661)

[**Table S6.** Classification performance, linden vs all other types	12](#_Toc239012662)

# 1. **Proton NMR spectrum**

**Figure S1**. 1D ^1^H spectrum of linden honey extract dissolved in D_2_O. The well-resolved signals of tiliaside are labelled with orange arrows, signals of new glycoside lindoside are labelled with green arrows. Linden honey specific regions obtained with PCA performed during NMR profiling of Slovenian honey are marked with grey bands.

# 2. **DOSY**

The SPE extract was spiked with the parent linden honey to reintroduce α-glucose and turanose, previously identified in it. Together with tiliaside, already present in the extract, these provided three internal standards with molecular weights of 180–506 g/mol. Diffusion coefficients for the three standards and for lindoside were obtained from a single DOSY experiment (D₂O, 298 K), so solvent, viscosity, and temperature were identical for all molecules. α-Glucose diffused at 4.2 × 10⁻¹⁰ m²/s, lower than in aqueous solution and consistent with the higher viscosity of the extract; the value agrees with that reported for proto-quercitol in honey (Gresley et al., 2012).

Molecular weights were estimated by internal calibration (Evans, 2020). This is preferable to direct application of the Stokes–Einstein equation,

*D = kT/(6πηr) (1)*

which is derived by considering a hard sphere at infinite dilution in a continuum fluid. For small molecules, both assumptions fail: solute and solvent molecules are of comparable size, so the friction term is not well described by a continuum treatment, and the equation relates D to hydrodynamic radius rather than to mass. Converting rH to MW requires the additional assumption of constant density, which predicts a cube-root dependence of D on MW that small molecules do not obey. Internal calibration avoids both problems. Because standards and analyte are measured in the same sample, the viscosity and temperature terms are common to all species and cancel, and the mass–size relationship is replaced by an empirically parameterized power law,

log D = −α·log MW + log K (2)

whose parameters were obtained from D and MW data for the standards in table S1. Three standards are typically used for internal calibration. The standards used are structurally similar to the analyte – saccharide and glycoside structures of comparable polarity and solvation – which matters because power-law parameters are specific to a class of compounds. Linear regression of log D on log MW resulted in

log D = −0.535 (±0.048) log MW − 8.17 (±0.12)	(3)

with R² = 0.992 and residual standard deviation 0.015 (n = 3; uncertainties are standard errors of the regression coefficients; Figure S2). The exponent corresponds to a fractal dimension δ = 1/α = 1.87 and lies close to the value of 0.500 expected for a Θ-solvent and to α = 0.49 reported for pullulan, with sucrose, cyclodextrins, dextrans, and a mannose-rich oligosaccharide also fitting the same calibration curve, water being close to a Θ-solvent for polysaccharides (Evans, 2020, and references therein). Back-calculation of the standards’ MW from equation 3 reproduced their nominal values to within 5.2 %; the larger relative error on the mass scale than on the diffusion scale reflects the approximately threefold amplification of uncertainty inherent in estimating MW from measured diffusion coefficients. Applying equation 3 to the diffusion coefficient of the lindoside signals gives an apparent molecular weight of 523 g/mol, deviating by 2.9 % from the value calculated for the proposed structure (504.2 g/mol), comparable to the 0.7–5 % accuracy typically reported for internal calibration.

**Table S1.** Molar mass and diffusion coefficients of internal standards used for the calibration curve and newly characterized lindoside. DOSY spectrum recorded on the same sample using solvent D_2_O, T = 25°C. Calculated M_W_ values obtained from the calibration curve are reported together with the deviation from their actual M_W_ in %.

| Molecule | *M_W_*  [g/mol] | *D*  [x 10^-10^ m^2^/s] | *M_W_, calc*  [g/mol] | *Deviation* |
| --- | --- | --- | --- | --- |
| α-glucose | 180.2 | 4.2 | 184 | +2.1% |
| turanose | 342.3 | 3.1 | 324 | -5.3% |
| tiliaside | 506.5 | 2.4 | 523 | +3.4% |
| lindoside | 504.2 | 2.4 | 523 | +3.0% |

**Figure S2.** Calibration curve relating molecular weight and diffusion coefficients of internal standards.

| logMw [g/mol] | logD [m2/s] |
| --- | --- |
| 2.25575 | -9.37675 |
| 2.53441 | -9.50864 |
| 2.70415 | -9.61979 |

| logMw [g/mol] | lindoside |
| --- | --- |
| 2.70603 | -9.61979 |

**Table S2.** Linest function results

| Parameter | value |
| --- | --- |
| Slope | -0.535±0.048 |
| Intercept | -8.16±0.12 |
| R^2^ | 0.992 |
| SE of regression, s | 0.015 |
| df | 1 |

# 3. **Full structural assignment**

**Figure S3.** 2D ^1^H-^13^C HMBC spectrum of linden honey extract dissolved in D_2_O.

**Figure S4.** Sugar region of H1' proton in the stacked 2D ^1^H-^13^C HSQC-TOCSY spectra of linden honey extract dissolved in D_2_O with arrayed mixing time (20–120 ms), incremented by 20. Carbon chemical shifts of sugar unit B are labelled with green lines.

**Figure S5.** 2D ^1^H-^13^C HSQC-TOCSY spectrum of linden honey extract dissolved in D_2_O with mixing time 100 ms.

**Figure S6.** Sugar region of stacked 1D ^1^H selective TOCSY spectra of linden honey extract dissolved in D_2_O recorded by selectively exciting the H1” proton and using various mixing times (20, 40, 60, 80 and 120 ms) with assigned protons of sugar unit C.

# 4. **Comparison of TOCSY-NOESY NMR spectra of lindoside and tiliaside compounds**

For comparison, a selective TOCSY-NOESY spectrum was also recorded for tiliaside (Figure S7). The H1' and H6b' protons, observed at different chemical shifts compared to those in lindoside, *δ_H_* 5.588 ppm and δ_H_ 3.804 ppm, respectively, were selectively excited. Comparison of the selective TOCSY-NOESY spectra of lindoside and tiliaside showed that their anomeric H1” protons of sugar unit C exhibit the same chemical shift and coupling constant.

**Figure S7.** 1D ^1^H selective TOCSY-NOESY spectra of linden honey extract dissolved in D_2_O with assignment of H5', H6a', H6b' and H1” protons of tiliaside (a) and lindoside (b). The chemical structures of tiliaside (c) and (d) lindoside are shown, with main structural difference highlighted by magenta arrows.

# 5. **NMR profiling information and quantification data for lindoside**

**Sample preparation and spectra acquisition**

To prepare samples for NMR experiments, approximately 1.5 g of each honey was dissolved in 5 mL of Milli-Q water. After complete dissolution by vortexing, 900 µL of the resulting solution was transferred into a new Eppendorf tube and 100 µL of wine buffer was added. To establish the desired pH of 3.1, controlled adjustments were made using either 1 M NaOH or HCl. One-dimensional ^1^H NMR spectra of the samples were acquired on a Bruker AVANCE NEO 400 MHz spectrometer (Ettlingen, Germany), and all NMR experiments were performed at 298.15 K. The pulse sequence noesygppr1d was used with the following parameters: 65,536 data points, 64 scans, spectral width of 20.5 ppm (8196.7 Hz), relaxation delay of 25 s, and a water saturation frequency of 1879.86 Hz (4.698 ppm).

**NMR spectral processing and statistical analysis**

Spectral preprocessing was done using MestreNova software. All NMR spectra were phased, baseline-corrected, and referenced to the TMSP signal. Each ^1^H NMR spectrum between δ 0.06 and 8.37 ppm was segmented into 238 bins (water and TMS signals excluded). The area under each binned region was integrated, and the integrated binned regions were normalized to a constant sum equal to 1. PCA and ANOVA were performed using the web-based metabolomic data processing tool MetaboAnalyst.

Geographically, Slovenia is divided into four regions: Alpine, Pannonian, Dinaric, and Mediterranean. The study included 81 honey samples. In the 2021 programme year there was no honey flow during task implementation, so samples from the 2019–2020 beekeeping season were included in the analysis; in the 2022 programme year, samples from the 2020–2021 harvest were analyzed, so that for each of the most common honey types characteristic of Slovenian origin, sampling covered two different years. Spruce honeydew flow has been very poor in recent years, so a sample from 2018 was analyzed. We analyzed sixteen samples of chestnut honey, fourteen of fir honey, twelve of multifloral honey, eleven of linden honey, eight each of buckwheat and forest honey, six of acacia honey, and four of oilseed rape honey, as listed in Table S3.

Statistical analysis was performed in R 4.6.1. Values were ln-transformed before analysis, as the variance was proportional to the mean (Levene's test on untransformed values: p = 0.041; on ln-transformed values: p = 0.158). Normality was assessed with the Shapiro–Wilk test within groups and on the model residuals. Differences between honey types were evaluated by one-way ANOVA followed by Tukey's HSD; Welch's ANOVA and the Kruskal–Wallis test were run in parallel to confirm that the conclusions did not depend on parametric assumptions. Classification performance of the marker was evaluated by setting the threshold as the geometric midpoint of the separation gap, with Clopper–Pearson confidence intervals for sensitivity and specificity, a bootstrap confidence interval for the threshold (10,000 resamples), and leave-one-out cross-validation. Effect sizes are reported as η² and Hedges' g.

**Table S3.** List of samples that were used for NMR profiling

| Honey type | Geographical region | Year | Sample code |
| --- | --- | --- | --- |
| Chestnut | Alpine | 2020 | K. MED 2021-1 |
| Chestnut | Alpine | 2020 | K. MED 2021-2 |
| Chestnut | Alpine | 2020 | K. MED 2021-19 |
| Chestnut | Alpine | 2020 | K. MED 2021-29 |
| Chestnut | Mediterranean | 2020 | K. MED 2021-20 |
| Chestnut | Pannonian | 2020 | K. MED 2021-27 |
| Chestnut | Pannonian | 2020 | K. MED 2021-30 |
| Chestnut | Pannonian | 2020 | K. MED 2021-38 |
| Chestnut | Alpine | 2021 | K. MED 2022-23 |
| Chestnut | Alpine | 2021 | K. MED 2022-24 |
| Chestnut | Alpine | 2021 | K. MED 2022-25 |
| Chestnut | Alpine | 2021 | K. MED 2022-26 |
| Chestnut | Alpine | 2021 | K. MED 2022-27 |
| Chestnut | Alpine | 2021 | K. MED 2022-28 |
| Chestnut | Alpine | 2021 | K. MED 2022-29 |
| Chestnut | Alpine | 2021 | K. MED 2022-30 |
| Multifloral | Alpine | 2020 | K. MED 2022-43 |
| Multifloral | Alpine | 2020 | K. MED 2022-47 |
| Multifloral | Dinaric | 2020 | K. MED 2022-45 |
| Multifloral | Dinaric | 2020 | K. MED 2022-46 |
| Multifloral | Pannonian | 2020 | K. MED 2022-44 |
| Multifloral | Pannonian | 2020 | K. MED 2022-48 |
| Multifloral | Alpine | 2021 | K. MED 2022-6 |
| Multifloral | Alpine | 2021 | K. MED 2022-12 |
| Multifloral | Dinaric | 2021 | K. MED 2021-39 |
| Multifloral | Dinaric | 2021 | K. MED 2022-8 |
| Multifloral | Mediterranean | 2021 | K. MED 2022-7 |
| Multifloral | Pannonian | 2021 | K. MED 2022-9 |
| Linden | Mediterranean | 2020 | K. MED 2021-13 |
| Linden | Mediterranean | 2020 | K. MED 2021-16 |
| Linden | Mediterranean | 2020 | K. MED 2021-17 |
| Linden | Mediterranean | 2020 | K. MED 2021-21 |
| Linden | Mediterranean | 2020 | K. MED 2021-22 |
| Linden | Mediterranean | 2020 | K. MED 2021-23 |
| Linden | Dinaric | 2021 | K. MED 2022-33 |
| Linden | Dinaric | 2021 | K. MED 2022-34 |
| Linden | Mediterranean | 2021 | K. MED 2022-31 |
| Linden | Mediterranean | 2021 | K. MED 2022-32 |
| Linden | Mediterranean | 2021 | K. MED 2022-35 |
| Buckwheat | Pannonian | 2020 | K. MED 2021-4 |
| Buckwheat | Pannonian | 2020 | K. MED 2021-5 |
| Buckwheat | Pannonian | 2020 | K. MED 2021-6 |
| Buckwheat | Pannonian | 2020 | K. MED 2021-7 |
| Buckwheat | Pannonian | 2020 | K. MED 2021-8 |
| Buckwheat | Pannonian | 2021 | K. MED 2022-1 |
| Buckwheat | Pannonian | 2021 | K. MED 2022-2 |
| Buckwheat | Pannonian | 2021 | K. MED 2022-3 |
| Acacia | Dinaric / Mediterranean | 2020 | K. MED 2022-5 |
| Acacia | Mediterranean | 2020 | K. MED 2021-9 |
| Acacia | Mediterranean | 2020 | K. MED 2021-14 |
| Acacia | Mediterranean | 2020 | K. MED 2021-15 |
| Acacia | Pannonian | 2020 | K. MED 2021-28 |
| Acacia | Pannonian | 2021 | K. MED 2022-4 |
| Oilseed rape | Pannonian | 2019 | K. MED 2021-34 |
| Oilseed rape | Pannonian | 2019 | K. MED 2021-35 |
| Oilseed rape | Pannonian | 2020 | K. MED 2021-32 |
| Oilseed rape | Pannonian | 2020 | K. MED 2021-33 |
| Honeydew (fir) | Dinaric | 2019 | K. MED 2021-10 |
| Honeydew (fir) | Dinaric | 2019 | K. MED 2021-11 |
| Honeydew (fir) | Dinaric | 2019 | K. MED 2021-36 |
| Honeydew (fir) | Mediterranean | 2019 | K. MED 2021-12 |
| Honeydew (fir) | Alpine | 2020 | K. MED 2022-18 |
| Honeydew (fir) | Alpine | 2021 | K. MED 2022-14 |
| Honeydew (fir) | Alpine | 2021 | K. MED 2022-17 |
| Honeydew (fir) | Alpine | 2021 | K. MED 2022-19 |
| Honeydew (fir) | Dinaric | 2021 | K. MED 2022-13 |
| Honeydew (fir) | Dinaric | 2021 | K. MED 2022-16 |
| Honeydew (fir) | Dinaric | 2021 | K. MED 2022-15 |
| Honeydew (fir) | Dinaric | 2021 | K. MED 2022-20 |
| Honeydew (fir) | Dinaric | 2021 | K. MED 2022-21 |
| Honeydew (fir) | Dinaric | 2021 | K. MED 2022-22 |
| Honeydew (forest) | Alpine | 2020 | K. MED 2021-18 |
| Honeydew (forest) | Alpine | 2020 | K. MED 2021-24 |
| Honeydew (forest) | Alpine | 2020 | K. MED 2021-25 |
| Honeydew (forest) | Alpine | 2020 | K. MED 2021-26 |
| Honeydew (forest) | Dinaric | 2020 | K. MED 2021-3 |
| Honeydew (forest) | Dinaric | 2020 | K. MED 2021-37 |
| Honeydew (forest) | Dinaric | 2021 | K. MED 2022-10 |
| Honeydew (forest) | Dinaric | 2021 | K. MED 2022-11 |
| Honeydew (spruce) | Dinaric | 2018 | K. MED 2021-31 |
| Honeydew (spruce) | Dinaric / Alpine | 2019 | K. MED 2021-40 |

**Table S4.** Relative integral (1.5369-1.5966 ppm) by honey type

| **Honey type** | **n** | **Median** | **IQR** | **Range** | **p vs linden** | **Ratio** | **Hedges' g** |
| --- | --- | --- | --- | --- | --- | --- | --- |
| Acacia | 6 | 1.77e-05 | 1.72e-05–2.33e-05 | 1.21e-05–2.58e-05 | <0.001 | 6.12 | 8.09 |
| Honeydew | 24 | 4.83e-05 | 4.55e-05–5.37e-05 | 3.84e-05–7.07e-05 | <0.001 | 2.29 | 5.13 |
| Oilseed rape | 4 | 5.10e-05 | 4.53e-05–5.64e-05 | 4.36e-05–5.71e-05 | <0.001 | 2.27 | 5.78 |
| Multifloral | 12 | 5.66e-05 | 4.17e-05–6.33e-05 | 3.34e-05–7.29e-05 | <0.001 | 2.22 | 3.64 |
| Chestnut | 16 | 6.22e-05 | 5.53e-05–6.83e-05 | 4.32e-05–7.26e-05 | <0.001 | 1.87 | 4.43 |
| Buckwheat | 8 | 6.87e-05 | 5.87e-05–7.17e-05 | 5.71e-05–7.70e-05 | <0.001 | 1.72 | 4.14 |
| Linden | 11 | 1.19e-04 | 1.03e-04–1.27e-04 | 8.80e-05–1.35e-04 | — | — | — |

**Table S5.** Overall tests

| **Test** | **Statistic** | **p** | **Effect size** |
| --- | --- | --- | --- |
| One-way ANOVA (ln) | F(6, 74) = 65.01 | < 0.001 | eta2 = 0.84 |
| Welch ANOVA (ln) | F(6, 20.3) = 51.30 | 3.3 x 10-11 | — |
| Kruskal-Wallis (raw) | chi2(6) = 51.71 | 2.1 x 10-9 | — |
| Mann-Whitney (linden vs rest) | W = 770 | 5.8 x 10-8 | g = 2.34 |
| Levene (raw) | F(6, 74) = 2.32 | 0.041 | — |
| Levene (ln) | F(6, 74) = 1.60 | 0.158 | — |

**Table S6.** Classification performance, linden vs all other types

| **Metric** | **Value** | **95% CI** |
| --- | --- | --- |
| Threshold | 8.232 x 10-5 | 7.99 - 8.92 x 10-5 (bootstrap) |
| Separation gap | 7.70 - 8.80 x 10-5 | — |
| Sensitivity | 11/11 = 100% | 71.5 - 100% |
| Specificity | 70/70 = 100% | 94.9 - 100% |
| ROC AUC | 1.000 | — |
| LOOCV accuracy | 81/81 = 100% | — |

The relative integral of the diagnostic region differed markedly between honey types (one-way ANOVA on ln-transformed values, F(6, 74) = 65.0, p < 0.001, η² = 0.84), with honey type accounting for 84 % of the variance. Linden honey exceeded every other type, with ratios of 1.7–6.1 and all six pairwise comparisons significant (Tukey HSD, p < 0.001; Table A). Welch’s ANOVA and the Kruskal–Wallis test yielded concordant results.

More importantly for authentication, the two groups did not overlap at any point: all eleven linden samples exceeded 8.80 × 10⁻⁵, whereas none of the remaining seventy samples exceeded 7.70 × 10⁻⁵. We emphasize that this describes complete separation within the present sample set; the threshold itself requires confirmation on independent samples before it can be applied predictively.

**Figure S8.** Region of the 2D HMBC spectrum of linden honey extract dissolved in D_2_O. Key long-range correlation signals characterizing the substituents attached to phenyl ring A are marked with green circles; the numbers correspond to the atom numbering shown in Figure 1. Green lines show correlations of the cluster of signals with similar chemical shifts, assigned to molecules structurally related to lindoside, whereas orange lines mark the corresponding set for tiliaside. Orange arrows show correlations between aglycone signals and additional sugar signals.

# 6. **LC–HRMS/MS analysis**

**Figure S9.** Extracted ion chromatogram (m/z 503.1764) of honey extract, diluted 500-fold with 20% MeOH. Major lindoside derivatives were found at the following retention times: 10.04 min, 11.04 min, and 11.16 min.

**Figure S10.** A representative lindoside derivative MS spectrum acquired at retention time 10.1 min. The spectrum was acquired in negative ionization mode.

**Figure S11.** MS/MS spectra of major lindoside derivatives found at 10.04 min (top), 11.04 min (middle), and 11.16 min (bottom). In all cases, a precursor ion at m/z 503.1764 was fragmented. The spectra were acquired in negative ionization mode.

# 7. **References**

Evans, R. (2020). The interpretation of small molecule diffusion coefficients: Quantitative use of diffusion-ordered NMR spectroscopy. In *Progress in Nuclear Magnetic Resonance Spectroscopy* (Vol. 117, pp. 33–69). Elsevier B.V. https://doi.org/10.1016/j.pnmrs.2019.11.002

Gresley, A. Le, Kenny, J., Cassar, C., Kelly, A., Sinclair, A., & Fielder, M. D. (2012). The application of high resolution diffusion NMR to the analysis of manuka honey. *Food Chemistry*, *135*(4), 2879–2886. https://doi.org/10.1016/j.foodchem.2012.07.072
